# Screening of insecticide resistance in *Aedes aegypti* populations collected from parishes in Eastern Jamaica

**DOI:** 10.1371/journal.pntd.0008490

**Published:** 2020-07-27

**Authors:** Sheena Francis, Towanna Campbell, Sashell McKenzie, Danisha Wright, Jervis Crawford, Trevann Hamilton, Sherine Huntley-Jones, Simone Spence, Allison Belemvire, Kristen Alavi, Carolina Torres Gutierrez

**Affiliations:** 1 Natural Products Institute, University of the West Indies, Mona, Jamaica; 2 Abt Associates, Kingston, Jamaica; 3 Zika AIRS Project Jamaica, Kingston, Jamaica; 4 Vector Control Unit, Ministry of Health and Wellness, Kingston, Jamaica; 5 Health Promotions and Protection, Ministry of Health and Wellness, Kingston, Jamaica; 6 Bureau for Global Health, Office of Infectious Disease, Malaria Division, United States Agency for International Development (USAID), Arlington, Virginia, United States of America; 7 Office of Infectious Diseases, United States Agency for International Development (USAID), Washington DC, United States of America; 8 International Development Division (IDD), Abt Associates, Rockville, United States of America; 9 Valent BioSciences, Libertyville, Illinois, United States of America; University of Queensland, AUSTRALIA

## Abstract

Owing to the increased reports in Aedes-borne diseases in the Caribbean and Latin America, the United States Agency for International Development assisted the Jamaican Ministry of Health and Wellness in conducting insecticide susceptibility tests on *Aedes aegypti* populations.

Sentinel sites were established in seven parishes of Jamaica (St. Catherine, Kingston and St. Andrew, St. Thomas, Portland, St. Mary and St. Ann) and *Aedes aegypti* eggs were collected, reared to adults per collected population and their susceptibility to varying pyrethroids and organophosphates were tested using the World Health Organization paper bioassays for these insecticides. The Centers for Disease Control and Prevention bottle bioassay was used to assess susceptibility to the carbamate, bendiocarb. The voltage gated sodium channel gene mutations *V1016I* and *I1011V*, normally associated with pyrethroid resistance, were also analysed.

The results showed that *Aedes aegypti* collected from all parishes exhibited resistance to pyrethroids at the following concentrations, permethrin 0.25–2.5%; deltamethrin 0.03–0.15%; lambda-cyhalothrin 0.03–0.3%; and etofenprox 0.5–2.5%. The insecticide deltamethrin at concentration 0.3% was the only pyrethroid tested that resulted in high mortality, 94.9 ± 0.34% knockdown within 1 hour of exposure and 98.95 ± 0.01% mortality (*p* <0.01) at 24 hours post exposure. The frequency of the voltage gated sodium channel gene mutation *V1016I* was high in the tested population, possibly accounting for the reduced sensitivity to pyrethroids. Organophosphate resistance was also observed in all populations tested. Mortality rates for 0.8% Malathion was 0.8 ± 0.70–60.68 ± 0.38% after 24 hour and 0.00–47.10 ± 3.02%, for pirimiphos-methyl 0.21%. Bendiocarb applied as 12.5 μg/ bottle resulted in mortality rates of 76.25 ± 4.30–100 ± 0.00% after 30 minutes of exposure.

The results showed that *Ae*. *aegypti* from the seven parishes analysed demonstrated resistance to the insecticides tested. Deltamethrin and bendiocarb at concentrations 0.3% and 12.5μg respectively, were considered most effective, causing high mortality in the local populations. Routine monitoring and evaluations of *Ae*. *aegypti* populations from the included parishes are recommended. Additionally, the study results represent the most comprehensive testing to date with local *Aedes aegypti* populations distributed across different parishes of Jamaica and should be useful to guide national and sub national strategies for vector control and surveillance.

## Author summary

The recent outbreaks of chikungunya, Zika and dengue viruses in the Caribbean and Latin America during 2013–2018, have motivated local health authorities in individual countries to seek international assistance from multiple agencies and adjust their vector management strategies in order to contain the transmission of Aedes-borne diseases. Countries within the region rely mainly on the use of commercially available insecticides to manage the mosquito populations and by extension, reduce the transmission of vector-borne diseases. However, this practice is stymied by the ability of these insects to develop resistance mechanisms to insecticides of frequent use. Many countries in the Caribbean and Latin America regions lack the facilities and supplies to conduct routine insecticide susceptibility testing. In an effort to ascertain knowledge on the presence of insecticide resistance in *Aedes aegypti* populations, and to determine which insecticide is more effective to reduce vector populations of Jamaica, we conducted an array of bioassays with three different types of insecticides (pyrethroid, organophosphate, carbamate) to use local evidence and inform public health authorities on vector control status and alternatives.

## Introduction

Each year, more than 284 million people are affected by dengue [1]. The main vector of the disease is *Aedes aegypti*, found ubiquitously throughout Jamaica and the wider Caribbean [2, 3]. The circulation of all four dengue serotypes in the region [4, 5], reports of increased dengue cases [6, 7], the recent introduction of other *Aedes*-borne diseases, such as chikungunya and Zika viruses [8], and the lack of specific treatments for the diseases have resulted in heightened urgency to improve vector management throughout the region. Additionally, the World Health Organization (WHO) [9] has been encouraging the health authorities in countries where mosquito-borne diseases are endemic to assess the susceptibility of the vectors to various insecticides and implement national insecticide resistance monitoring strategies. In response to the Zika outbreak and to a recognized lack of current nationwide insecticide susceptibility analysis, the Ministry of Health and Wellness (MOHW) of Jamaica, partnered with the United States Agency for International Development (USAID) under the Zika AIRS Project (ZAP), to conduct island-wide interventions to monitor and control populations of the mosquito vector *Ae*. *aegypti*. A key element of this collaboration was the assessment of the susceptibility status of this mosquito vector to commonly used insecticides.

The use of chemical insecticides for community spraying is the main method to control the adult *Ae*. *aegypti* populations. However, this method is limited by the ability of insects to develop resistance to the chemicals of frequent use [10]. Many countries have reported resistance in mosquitoes to common classes of insecticide, pyrethroids, organophosphates or carbamates, while others have reported cross-resistance to one or more pesticides [11–13]. The vector control programme of Jamaica has utilized organophosphates [2] and most recently pyrethroids [14] to reduce *Ae*. *aegypti* densities. Organophosphates and carbamates are acetylcholinesterase inhibitors, toxins that trigger prolonged neurological synapses in mosquitoes, eventually resulting in paralysis, while pyrethroids on the other hand, are fast-acting neurotoxins. Unlike organophosphates and carbamates, their mode of action prevents the closure of protein-voltage-gated sodium channels (vgsc) on neurons, causing excess excitation, and eventually paralysis. Alterations in the specific binding site of a neurotoxin in mosquitoes can confer resistance to the insecticide [14–16]. Mosquitoes may display one or more of these modes of resistance, making them less susceptible to insecticides [12]. The level and type of resistance developed by mosquito populations are usually discerned through bioassays and biomolecular tests [17]. Depending on the level of resistance, bombarding the target site with copious amounts of the toxic chemical can restore susceptibility by ensuring that enough chemical is delivered to the site prior to total chemical decomposition [18], which may have long term implication in successful vector management.

Resistance may vary across different geolocations despite proximity [19]. Effective environmental management of the vector, which is often complementary to the use of chemical insecticides, can be thwarted by unplanned urbanization, inconsistent water supply to human dwellings, water storage practices, improper waste disposal, and the lack of community engagement in developing countries [20]. Though multiple studies exist on insecticide resistance throughout Latin America [21–23] and the French Caribbean [24], only a few studies investigating resistance in *Ae*. *aegypti* populations from one or two parishes of Jamaica are available [3, 14, 25] Studies focused on the molecular basis of insecticide resistance in vector populations of several countries in the Americas, have showed that resistance to an insecticide, within a given population, may vary depending on the presence and prevalence of genes associated with this characteristic, as well as the presence of selective stressors for resistance in the environment [14, 19, 22, 26–29].

The aims of this study were to determine the susceptibility of *Ae*. *aegypti* populations from Jamaica to various adulticides and provide evidence on the status of mosquito vector susceptibility to the three commercially available insecticides (pyrethroids, organophosphates and carbamates). The overall goal was to support the local health authorities and foster evidence-based decisions for *Aedes* management strategies.

## Methods and materials

### Study Site

**Fig 1:**
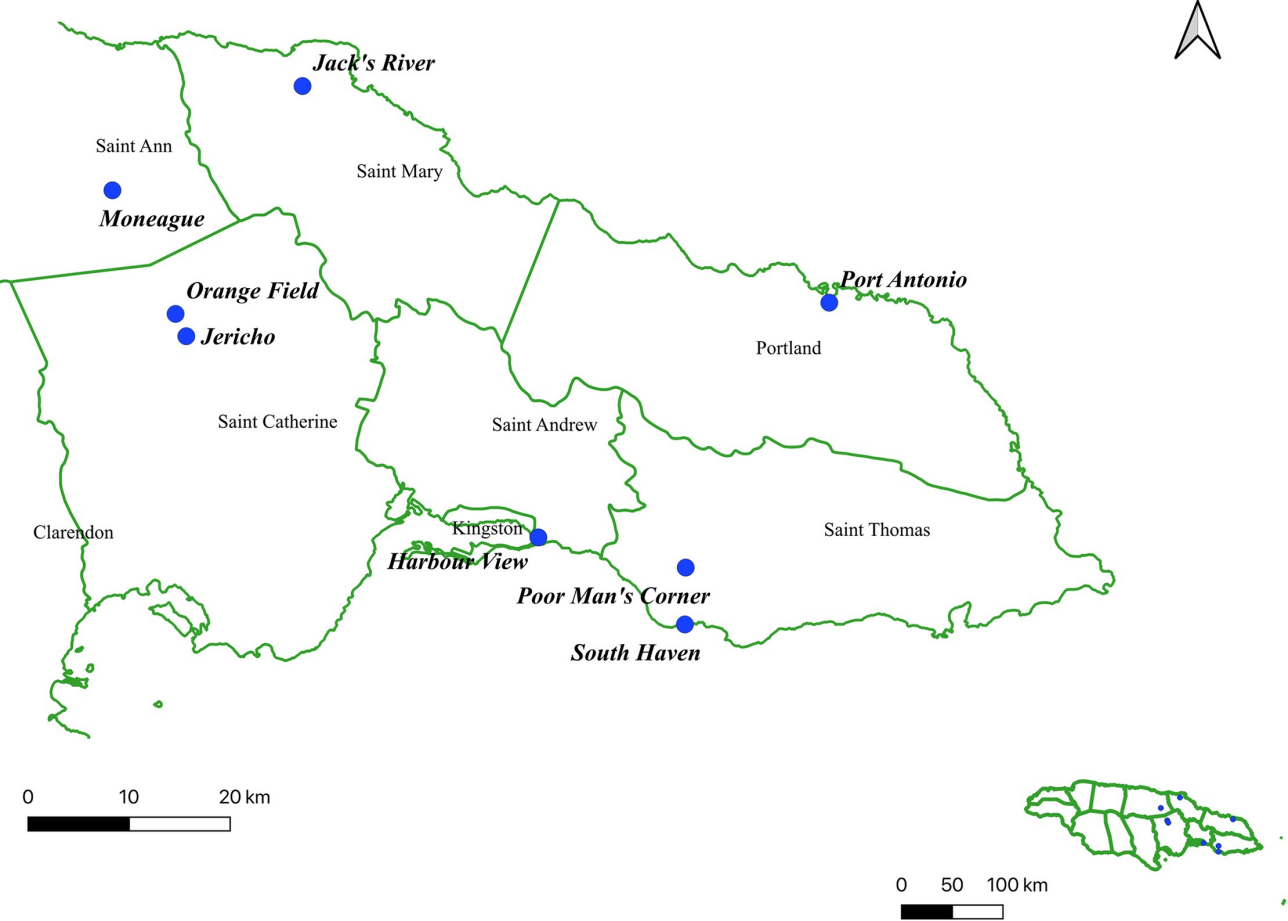
Map showing the collection areas in Jamaica. The above map of Jamaica was generated by using QGIS (version 3.10). The areas highlighted show where sentinel sites were established in St. Catherine, Kingston and St. Andrew (KSA), St. Thomas, Portland, St. Mary and St. Ann.

This study was developed in Jamaica, in the eastern parishes of the island: St. Catherine, Kingston & St. Andrew (KSA), St. Thomas, Portland, St. Mary, and St. Ann. The specific locations were selected in agreement with the MOHW mainly based on the epidemiological records of 2015 and 2016. Parishes with the highest incidence of Zika cases and classified as the most populated and accessible in the eastern territory were included in the study [30]. Specific locations in the eastern parishes, here designated as sentinel sites, were selected for entomological surveillance and sampling of biological material: Jacks River and Gueddes Town in the parish of St. Mary; Moneague in the parish of St. Ann; Jericho and Orangefield in the parish of St. Catherine; Harbour View in Kingston; South Haven and Poor Man’s Corner in the parish of St. Thomas. In the parish of Portland, the field collection took place in the municipality of Port Antonio (Fig 1).

### Materials

WHO susceptibility test kit tubes, with impregnated papers, pyrethroid (PY) control in Silicone oil; permethrin 0.25%, 1.25%, 2.5%; deltamethrin 0.03%, 0.15%, 0.3%; lambda-cyhalothrin 0.03%, 0.15%, 0.3%; etofenprox 0.5%, 2.5%; organophosphates (OP)/ carbamate (Carb) control in olive oil; malathion 0.8%; pirimiphos-methyl 0.21% were purchased from Universiti Sains Malaysia (USM), Vector Control Research Unit, Infotech (Pinang, Malaysia), which is the only entity approved by the World Health Organization (WHO) to supply insecticide kits (mosquito diagnostic test kit WHO/VBC/81.806) and materials for regular entomological surveillance in public health. Analytical grade bendiocarb (> 95%; Sigma, Aldrich, Ca. USA), and (99.7%; Sigma, Aldrich, Ca. USA) acetone were also employed. Black plastic cups and pellon paper, which were used as ovitraps (described below) and egg papers respectively, were locally sourced in Kingston, Jamaica.

### Ovitraps & field collection

The biological material employed in this study was obtained through a continuous entomological monitoring system, deployed by ZAP in collaboration with the MOHW of Jamaica. ZAP’s support to the MOHW included teams of field workers distributed in different parishes of Jamaica, consistently implementing households visits and data collection on *Aedes aegypti* populations. The surveillance system established by ZAP selected a number of sentinel sites for frequent visits and deployment of ovitraps to collect *Ae*. *aegypti* eggs. Sentinel sites were established in 100 homes in each of the parishes on the eastern half of the island: St. Catherine, Kingston & St. Andrew (KSA), St. Thomas, Portland, St. Mary, and St. Ann. Field activities in the parishes of Kingston and St. Andrew were regularly managed under one umbrella within the MOHW vector program in Jamaica. Therefore, to maintain the zoning, monitoring and evaluation surveillance of the MOHW, ovitraps set throughout the parishes of Kingston and St. Andrew were described as being derived from KSA. The ovitrap surveillance was conducted weekly, and once the field teams visited the homes selected for this process, all pellon papers containing mosquito eggs were transported to a laboratory for analysis. All laboratory activities were conducted at the Mosquito Control and Research Unit (MCRU) Insectary, funded by ZAP and The University of the West Indies (UWI).

Prior to the operational activities, local communities within each parish were engaged by ZAP and the local health authorities. The community members were informed about the intended activities planned by the project. All householders who participated in the study approved the operational activities of ZAP and granted permission to their property on a weekly basis between September 17 to December 14, 2018, to collect samples and record information.

Black oviposition cups (450 ml capacity x 14.5 cm tall) lined with pellon paper (7 cm wide) and filled with 50 ml water containing 5 g of instant yeast (Lesaffre, France) were set up through the period of September 17^th^ 2018 to December 7^th^ 2018. Yeast was used as a mosquito attractant. The traps were replaced weekly and papers were collected from each parish and brought to the MCRU insectary. Once egg papers received at the insectary, these were air dried and assessed for the presence of *Ae*. *aegypti* eggs; a sample of papers was periodically set to hatch for susceptibility bioassays.

### Mosquito rearing

The egg papers (25 per parish) were submerged in acrylic containers (37.5 × 25 × 15 cm) with 3 L of water with 0.1 g of instant yeast (Lesaffre, France). To increase egg hatching, we replicated the media and methods described by Zheng et al [31]. Tap water was collected, allowed to stand for a minimum of 48 hours, boiled, covered and allowed to acclimate to room temperature for 24 hours prior to adding the yeast and egg papers. The containers with the egg papers were then covered until larvae emergence was observed. The larvae were maintained under standard rearing conditions at 25 ± 2 ^0^C temperature, 75 ± 5% relative humidity and 12:12 h light: dark photoperiod. The larvae were fed on a diet of ground cat food (Friskies). Pupae were placed in emerging chambers (Bioquip, Ca., USA) until the eclosion to adults and then transferred to 30 x 30 x 30 cm acrylic cages (Bug Dorms, Bioquip, Ca., USA).

Rockefeller laboratory strain (*Ae*. *aegypti*) eggs were initially donated from the ZAP Dominican Republic project (originally donated by the Centers for Disease Control and Prevention (CDC), Atlanta). This strain was used as the insecticide-susceptible reference strain for mortality comparison with the Jamaican wild populations. The strain was reared in isolation at the MCRU insectary, Jamaica, under the local insectary conditions described above.

Bioassays were conducted using 2–5 day old female mosquitoes that were briefly anaesthetized on ice and identified to species level prior to testing.

### WHO bioassays

Using the WHO method [9], the susceptibility of adult mosquitoes from the eastern parishes of Jamaica to the following insecticides and concentrations were examined: pyrethroids–permethrin 0.25%; deltamethrin 0.03%; lambda-cyhalothrin 0.03%; etofenprox 0.5%; and organophosphates–malathion 0.8% and pirimiphos-methyl 0.21%. Each kit had two specially marked tubes: one designated for the treatment paper (the exposure tube), the other designated for holding/ observation. Tubes marked with red dots were designated for the insecticide treated paper, while those with yellow dots were designated for the control paper (pyrethroid control paper impregnated with silicone oil, and organophosphate control paper impregnated with olive oil); all holding tubes had green dots.

A sample of 20–25 *Ae*. *aegypti* female mosquitoes per population were placed in the holding tubes where they were allowed to recover for five minutes prior to transferring them to the affixed exposure tubes. Each test was conducted along with a control. During testing, mosquitoes were observed up to one hour and knockdown was recorded. After the time had elapsed, the mosquitoes were returned to the holding tube and the exposure-tube removed. The mosquitoes remained in the holding tube for a further period of 24 hours and fed 10% sugar solution and final mortality was recorded. All bioassays were conducted in quadruplets, 70–111 females per population.

### WHO intensity bioassays

The WHO intensity assays employed in this study corresponded to higher concentrations of pyrethroids. The following intensity concentrations were used: pyrethroids at 5x and 10x –permethrin 1.25%, 2.5%; deltamethrin 0.15%, 0.3%; lambda-cyhalothrin 0.15%, 0.3% and etofenprox 2.5% papers were conducted [9]. Groups of 22–25 *Ae*. *aegypti* females per population were placed in the holding tubes where they were allowed to recover for 5 minutes prior to transferring them to the affixed exposure tubes. Each test was conducted along with a control. During testing, mosquitoes were observed up to one hour and knockdown was recorded. After the time had elapsed, the mosquitoes were returned to the holding tube and the exposure-tube removed. The mosquitoes remained in the holding tube for a further period of 24 hours and fed 10% sugar solution and final mortality was recorded. All bioassays were conducted in quadruplets.

### CDC bottle bioassays

In order to assess the susceptibility *Ae*. *aegypti* populations from the eastern parishes of Jamaica, to the carbamate bendiocarb, the method developed by the CDC [12] was used. Samples of 18–27 female mosquitoes were introduced into 250 ml Wheaton bottles previously coated with 12.5μg/bottle bendiocarb or acetone for the control. Mortality was recorded every 15 minutes up to 2 hours and then at 24 hours. All bioassays along with their control were conducted in quadruplets–a total of 75–100 female mosquitoes per population per assay. *Ae*. *aegypti* females were sorted and allowed to recover in a 50 ml falcon tube, where the opening was fitted with a cotton ball, prior to adding mosquitoes to the Wheaton bottles. All mosquitoes were active prior to their transfer to the treatment bottles.

### DNA isolation and SNP genotyping

The pyrethroid WHO susceptibility assays with permethrin 0.25%; deltamethrin 0.03%; and lambda-cyhalothrin 0.03% were repeated on *Ae*. *aegypti* (n = 75 per susceptibility assay) collected from St. Catherine, Jamaica. Mosquitoes from the tests were placed in 15 ml Eppendorf tubes per test (n = 20–25 *Ae*. *aegypti*), tubes were labelled with the test compound as well as either dead or alive to denote the status of the mosquitoes after the completion of the bioassays. Each tube was previously packed with silica gel beads and cotton prior to adding mosquitoes. The samples were shipped to the Lobo Lab, University of Notre Dame, Notre Dame, IN 46556 for genetic analysis.

All extractions were completed using Rapid Alkaline DNA Extraction protocol [32]. DNA was suspended in final volumes of 1,000 μL containing 0.2 N NaOH and 1 M Tris-HCl, pH 8.0. Single nucleotide polymorphism (SNP) identification for replacements in the voltage-dependent sodium channel gene (*Val1016*, *and Ile1011*, from here on referred to as *V1016I* and *I1011V* respectively) [10, 33] in *Ae*. *aegypti* were assessed. Control mosquitoes with and without insecticide resistant alleles, used for *V1016I* diagnosis were the New Orleans and the Liverpool strains of *Ae*. *aegypti* respectively. Amplification was performed in 25 μl volumes in 96-well PCR plates (Dot Scientific) in a Mastercycler Gradient thermocycler (Eppendorf). Each reaction contained 1X Taq buffer (50 mM KCl, 10 mM Tris pH 9.0, 0.1% Triton X), 1.5 mM MgCl_2,_ 200 μM dNTPs, 5 pmoles of each primer (Table 1), 1 unit of Taq DNA polymerase, and 3 μl of genomic DNA. PCR products were size fractionated by electrophoresis in 4% agarose gels stained with SybrSafe (Invitrogen) and visualized under UV light. Assays performed by Dr. Neil Lobo at the Department of Biological Sciences, Eck Institute for Global Health, University of Notre Dame, Notre Dame, IN, United States.

**Table 1:** List of Primer sequences used in detecting allele specific *kdr* mutations. F and R denote the forward and reverse primers respectively, while wt and mut signify wild-type and mutant sequence respectively.

*kdr* mutations	Primer Sequences	Fragment Size (bp)	References
V1016I	Isol016 F mut: GCGGGCACAAATTGTTTCCCACCCGCACTGA	82	[33]
	Isol016 R: GGATGAACCGAAATTGGACAAAAGC		
	Vall016 F wt: GCGGGCAGGGCGGCGGGGGCGGGGCCACAAATTGTTTCCCACCCGCACCGG	102	
I1011V	Isol011R wt: GCGGGCTACTTACTACTAGATTTCCAAT	84	[34]
	Vall011F: ATTGTATGCTTGTGGGTGACG		
	Vall011R mut: GCGGGCAGGGCGGCGGGGGCGGGGCCTACTTACTACTAGATTTCCGAC	104	

### Data analysis

The data are presented as mean ± 95% confidence interval (*95% CI)* per population. Abbott’s formula [35] was used to correct the mortality rate in each treated group when necessary.

% Corrected Mortality = ((T–C)/ (100 –C)) x 100; whereby T is equal to the total percent mortality in the treated group, and C is equal to the percent mortality in the control group, providing that the control mortality was ≤ 20%. The mortality in the control group for all bioassays per parish was less than 20%.

All statistical analyses were completed using SPSS for Windows (version 17.0). One-way analysis of variance (ANOVA) and *Posthoc Tukey test* were used to analyze significant differences (*p*<0.05) in susceptibility to insecticide between the Rockefeller strain and tested Jamaican populations, where possible. R (version 3.6.2) was used to calculate confidence intervals.

## Results

This study was undertaken with the aim of examining the susceptibility status of *Ae*. *aegypti* mosquito populations in seven parishes of Jamaica. The results showed evidence of high levels of resistance against six commonly used insecticides. In comparison to the Rockefeller strain, all populations from Jamaica sampled in this study demonstrated resistance to the pyrethroids, permethrin, deltamethrin, lambda-cyhalothrin and etofenprox, as well as the organophosphates, malathion and pirimiphos-methyl with the highest mortality displayed at 68.17 ± 3.26% over 24-hours exposure. However, the tested populations appeared responsive to the carbamate, bendiocarb. The differences in the levels of resistance for each insecticide tested were further compared between the populations.

### Impact by pyrethroids

**Fig 2:**
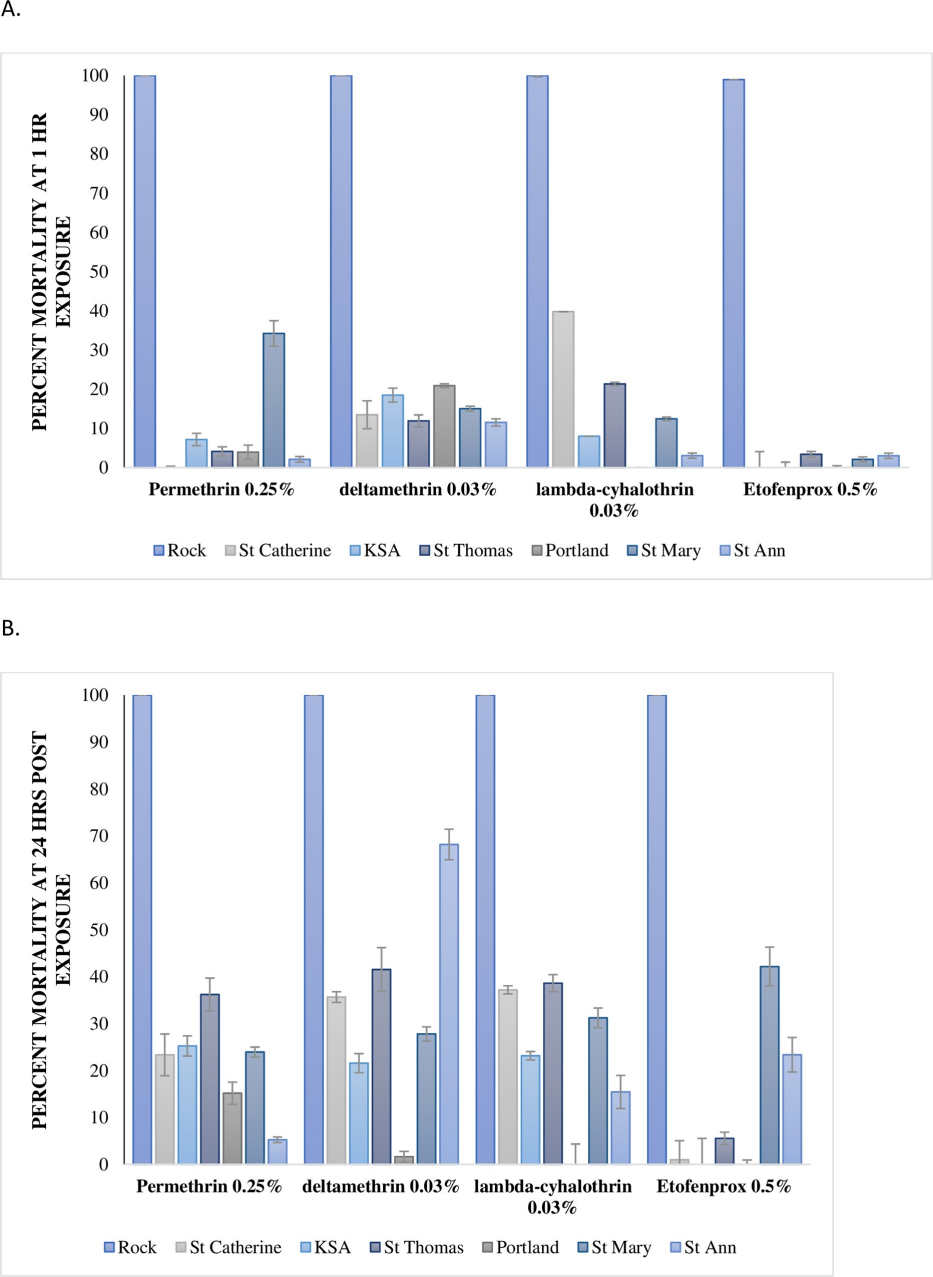
Percentage mortality of *Ae*. *Aegypti* exposed to baseline concentrations of several pyrethroids. The effect of the pyrethroids at baseline concentration (0.25% permethrin; 0.03% deltamethrin; 0.03% lambda-cyhalothrin and 0.5%, etofenprox) at (A)1–hour and (B) 24 hours on 2–5 day old *Ae*. *aegypti* F_0_ females from the eastern parishes of Jamaica, compared to Rockefeller, laboratory susceptible strain. The data are summarized as mean ± 95% CI.

Fig 2 depicts the impact (after one hour and 24 hours) of each tested pesticide on the levels of mortality by adult *Ae*. *aegypti* mosquitoes from the seven parishes under study. As can be seen in Fig 2A, one hour exposure was ineffective in eliciting any significant mortality in all populations, with the highest rate being observed at 39.8 ± 4.06% in the St. Catherine population by lambda cyhalothrin. When observed at 24 hours post exposure, mortality was below a maximum of 70%.

The percent mortality after 24 hour post-exposure to 0.25% permethrin ranged between 5.24 ± 0.59–36.19 ± 3.51% in *Ae*. *aegypti* F_0_ females for all populations, where the St. Ann population (*p* < 0.01) was the least affected by the pesticide. However, 24 hours post-exposure to 0.03% deltamethrin resulted in mortality ranging from 1.63 ± 1.14–68.17 ± 3.26%, with the St. Ann population being the most affected (*p* < 0.01). The results at 24 hours post-exposure to 0.03% lambda-cyhalothrin were not effective either, with mortality ranging from 15.43 ± 3.68–38.61 ± 1.32%. Similarly, post-exposure to 0.5% etofenprox caused 0.00 ± 0.89–42.14 ± 2.10% mortality with the insecticide having negligible effect on St. Catherine (0.98 ± 4.4%), KSA (0.00 ± 0.89%), St. Thomas (5.5 ± 1.81%) and Portland (0.00 ± 4.34%) populations (*p* < 0.01).

**Fig 3:**
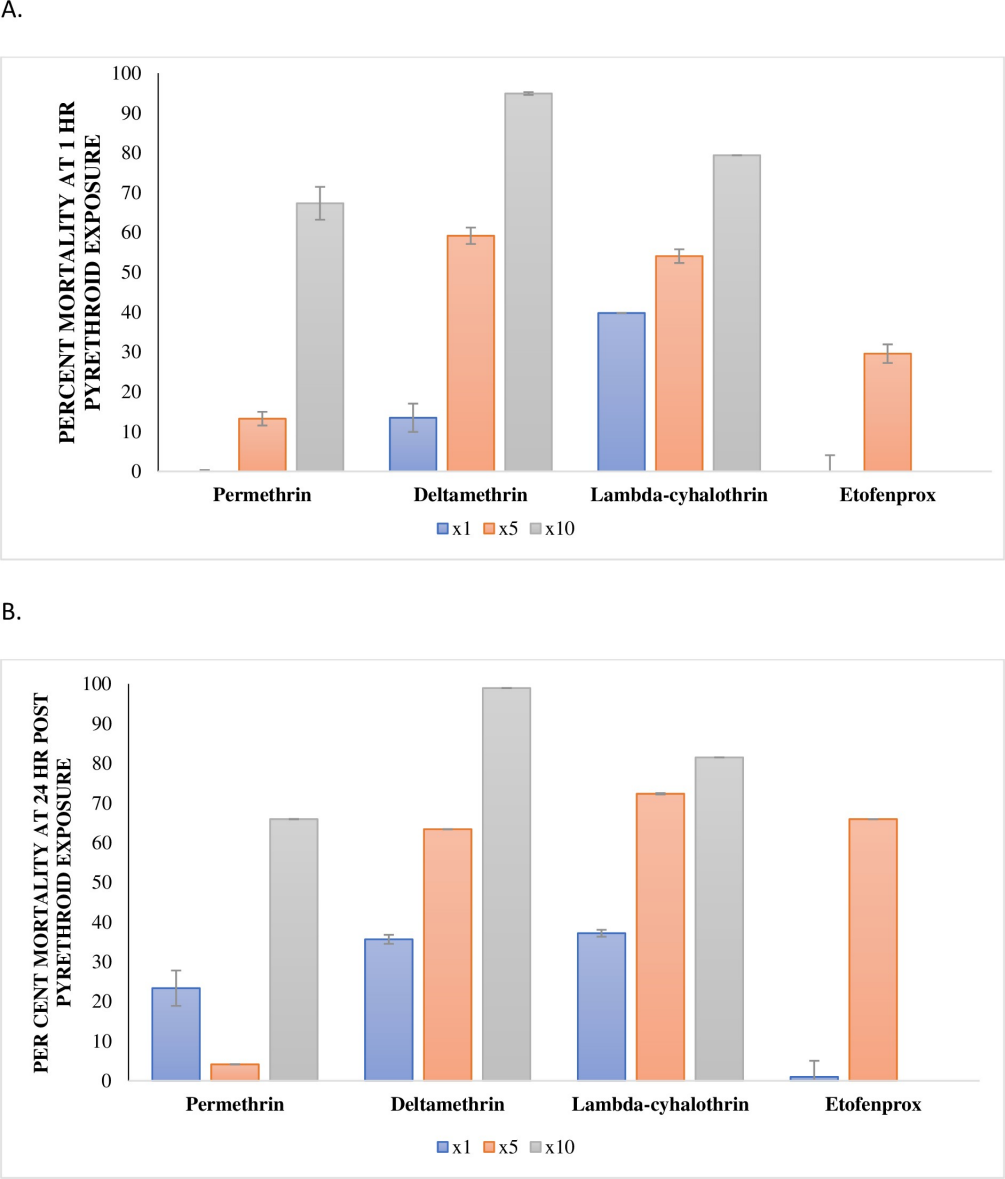
Percentage mortality of St. Catherine *Ae*. *aegypti* population exposed to 1x, 5x or 10x Concentrations of Several Pyrethroids. The effect of 0.25%, 1.25% and 2.5% permethrin; 0.03%, 0.15%; 0.3% deltamethrin; 0.03%, 0.15% and 0.3% lambda-cyhalothrin; and 0.5% and 2.5% etofenprox at (A) 1 hour and (B) 24 hours on 2–5 day old *Ae*. *aegypti* F_0_ females from St. Catherine. The data are summarized as mean ± 95% CI.

There were no significant differences between the pyrethroids tested and mortality rates amongst the populations, with the exception of St. Ann. As such, one population, St. Catherine, was selected for the WHO resistance intensity assays as well as for *kdr* mutation assays. The results of the WHO resistance intensity assays on the St. Catherine population are summarized in Fig 3. As can be seen in Fig 3, a concentration dependent effectivity was observed for the pesticides tested with highest effectivity rendered by 0.3% deltamethrin.

The effects of the pyrethroids, permethrin, deltamethrin, lambda-cyhalothrin and etofenprox at 5x or 10x concentration were assessed on samples collected from St. Catherine and the results compared to that of their 1x concentration. Exposure to 0.3% deltamethrin, appeared most effective, resulting in mortality greater than 98% at 24 hours post exposure. Exposure to either 1.25% or 2.5% permethrin resulted in 4.14 ± 0.03 and 65.93 ± 0.05% mortality, respectively after 24 hours post exposure. Exposure to lambda-cyhalothrin caused less than 85% mortality, even at its 10x (0.3%) concentration after 24 hours post exposure. Only the 5x (0.5%) etofenprox was used in this study, 24 hours post exposure resulted in only 65.93 ± 0.05% mortality of *Ae*. *aegypti*.

### Effects by organophosphates

**Fig 4:**
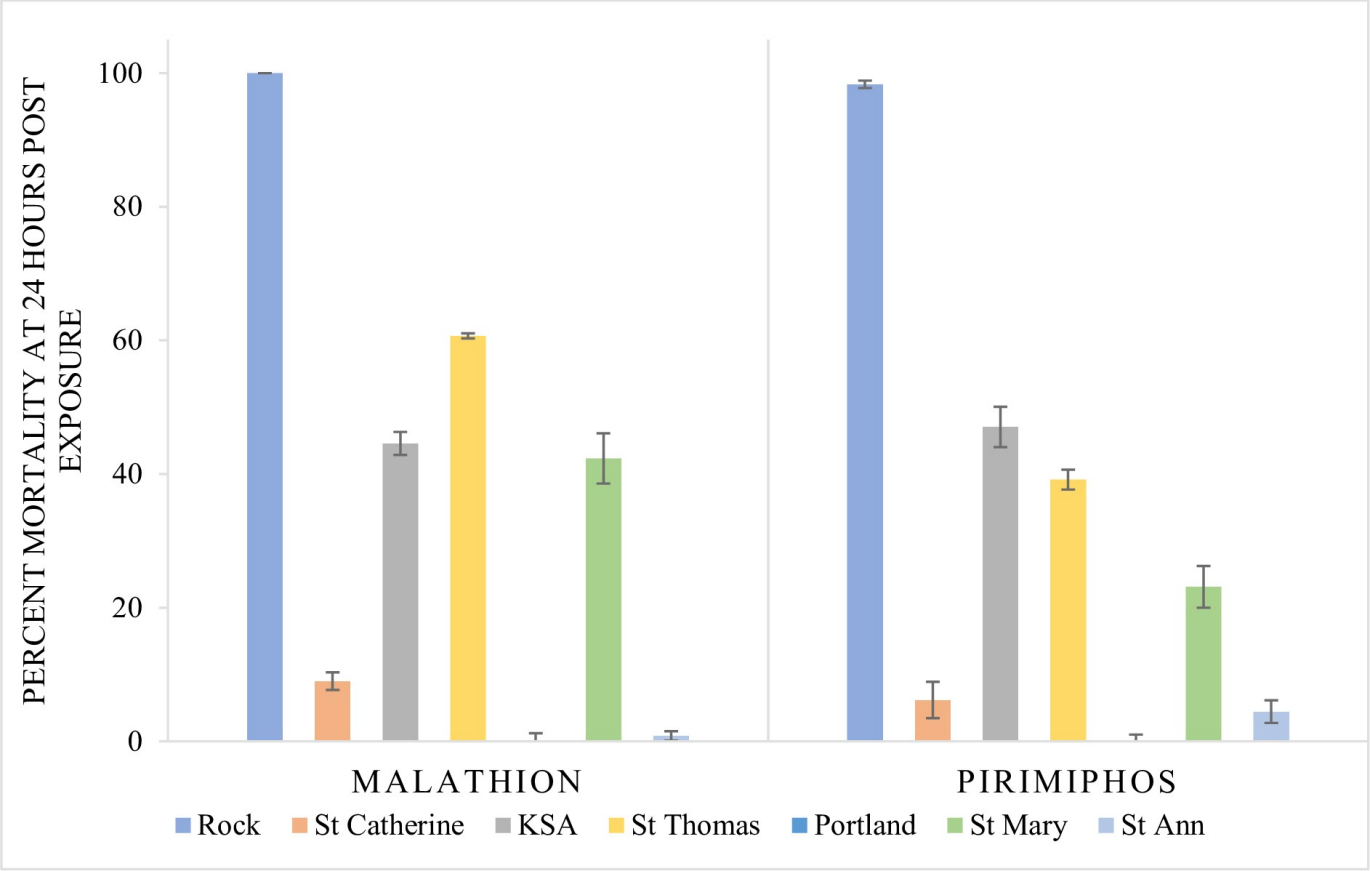
Percentage Mortality of *Ae*. *aegypti* Exposed to Baseline Concentrations of 0.8% Malathion or 0.21% Pirimiphos After 24-Hour Exposure. The effect of 0.8% malathion or 0.21% pirimiphos at 24 hours on 2–5 day old *Aedes aegypti* females reared from eggs collected from the eastern parishes of Jamaica. The data are summarized as mean ± 95% CI.

The effects of the organophosphates 0.8% Malathion or 0.21% pirimiphos-methyl after 1-hour exposure were considered negligible (See S1 Table). The mortality from 0.8% malathion after 24 hours post-exposure (Fig 4) ranged between 0.8–60.68%, with mosquitoes from St. Catherine (9.00 ± 1.32%) and St. Ann (0.8 ± 0.7%) being the least affected (*p* < 0.01). Similarly, 0.21% pirimiphos-methyl at 24 hours post-exposure was negligible, failing to cause mortality greater than 50% in the test populations, in comparison to the Rockefeller control, where Malathion or pirimiphos-methyl at the given concentrations, resulted in 95–100% mortality within 1 hr and 100% mortality at 24 hrs.

### Effect of carbamates

**Fig 5:**
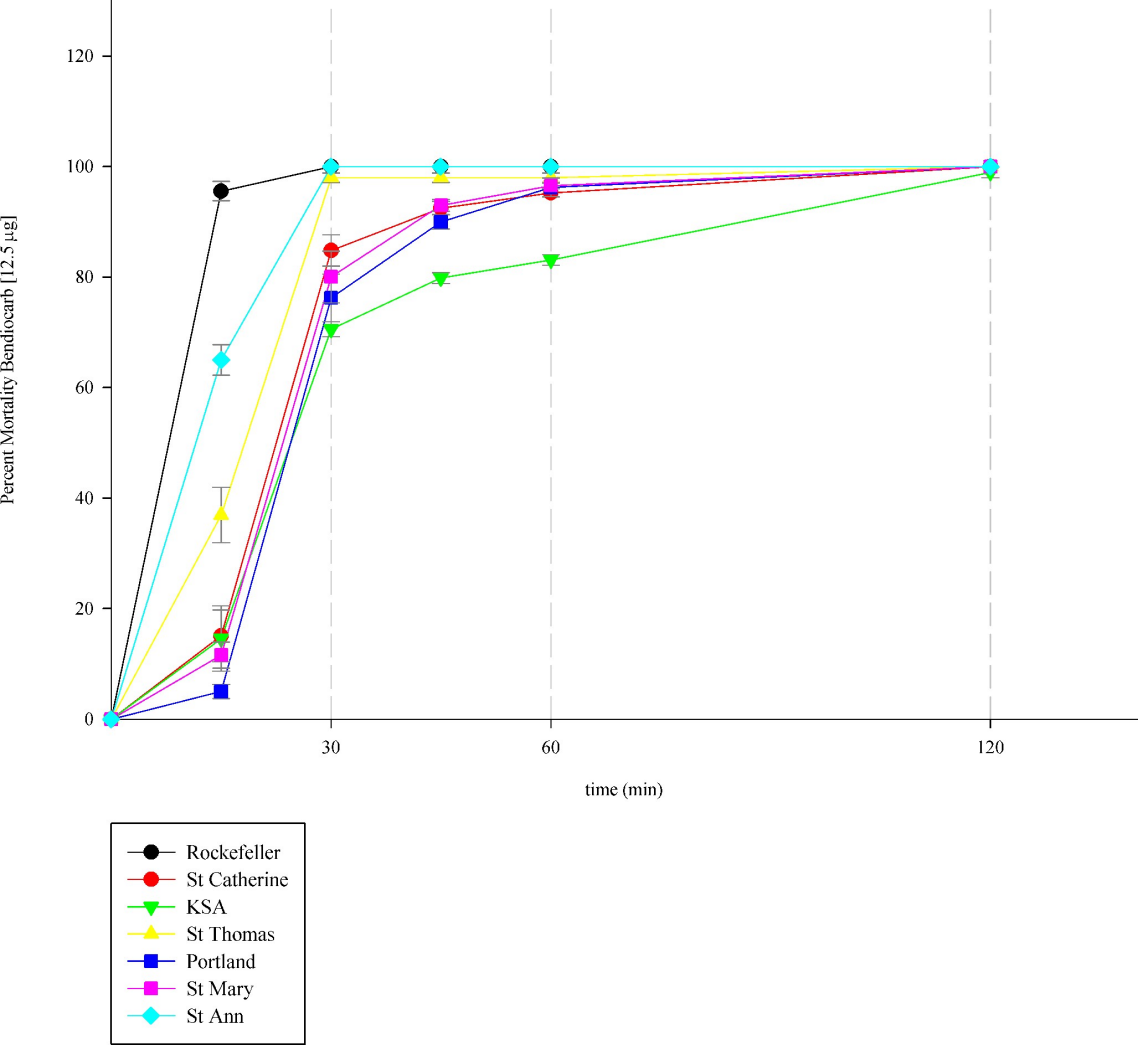
Bendiocarb susceptibility. The effect of bendiocarb on *Ae*. *aegypti* females from the eastern parishes of Jamaica using the CDC diagnostic dose of 12.5μg/bottle. Demarcation lines on the graph show the effect of bendiocarb at the CDC diagnostic time of 30 minutes, at 60 minutes, and at 120 minutes. The data are summarized as mean 95% CI.

Bendiocarb at 12.5μg resulted in mortality of 84.81 ± 2.82 for St. Catherine, 79.96 ± 1.36% for KSA, 98.00 ± 0.87 for St. Thomas, 76.25 ± 4.30% for Portland, 80.03 ± 4.70 for St. Mary, and 100 ± 0.00 for St. Ann, with no significant differences in mortality between the treated populations and the susceptible Rockefeller *p* = 0.10 (Fig 5). For the Rockefeller population, 100 ± 0.00% mortality was observed at 30 mins exposure.

### Mechanistic studies

The genotype mutation frequencies typically associated with knockdown resistance in the voltage gated sodium channel gene were examined to gauge mechanisms underlying the observed tolerance to pyrethroids in these adult populations. Results displayed in Table 2 reveal high frequencies for *V1016I* mutations, confirming *kdr* pathway as a means for the developed resistance to permethrin.

**Table 2:** Kdr Genotype Frequencies in Live Field Adult F0 Ae. aegypti Collected from St. Catherine, Jamaica.

	Mutation	Genotype			N
		Val/Val	Val/Ile	Ile/Ile	
**Total**	**V1016I**	**10**	**32**	**27**	**69**
					
	**Permethrin**	1	8	16	
	**Deltamethrin**	3	12	5	
	**Lambda cyhalothrin**	6	12	6	

Pyrethroid susceptibility assays were conducted on populations collected from St. Catherine Jamaica. The results shown are from mosquitoes that survived after the WHO screening with either 0.25% permethrin, 0.03% deltamethrin or 0.03% lambda cyhalothrin (all mosquitoes survived during this set of screening, as such results were only obtained for live mosquitoes). DNA was extracted from a total, N = 69 mosquitoes (n = 25 live *Ae*. *aegypti* for permethrin, n = 20 deltamethrin and n = 24 lambda-cyhalothrin) were assessed for *V1016I and I1011V*. All samples tested had the wild-type allele for *I1011* (see S2 Table). The 4% agarose gel was visualized by UV light after staining with SybrSafe.

## Discussion

The current study presents an updated and comprehensive profile on the state of insecticide susceptibility in Jamaican *Ae*. *aegypti* populations to common adulticides used in vector abatement programs. While previous studies on insecticide resistance in Jamaica focused on one or two classes of pesticide, selecting samples from one parish, this study examined the susceptibility of adult populations of *Ae*. *aegypti* from seven parishes (St. Catherine, Kingston and St. Andrew (KSA), St. Thomas, Portland, St. Mary, and St. Ann).

From the study, the Jamaican populations tested demonstrated resistance to pyrethroids and organophosphates. Deltamethrin was the only pyrethroid that caused high mean mortality in one of the six established populations. The highest mortality observed for deltamethrin 0.03% was 68.17 ± 3.26% in the St. Ann population. Surprisingly, this was the highest mortality (*p* <0.01) range for all pyrethroids tested at the 1x concentrations (see Fig 2B). This is in stark contrast to the laboratory susceptible Rockefeller population, where all pyrethroids tested at the 1x diagnostic concentrations resulted in 100% mortality within one hour of exposure (Fig 2A). According to the WHO guidelines [36], resistance is determined when mortality at the recommended dosage and time results in less than 90% mortality. The poor knockdown effect and the overall low toxicity caused by the pyrethroids tested on all the *Ae*. *aegypti* populations from Jamaica is evidence of strong resistance to these insecticides. Pyrethroids are fast acting [37] and are generally found in household insecticides [38, 39] as well as in products that guard against ectoparasites in domestic animals [40]. Pyrethroids were recently introduced as a vector management tool against mosquitoes by the MOHW, Jamaica (*pers comm*. Huntley-Jones, MOHW). As such, *Ae*. *aegypti* resistance to pyrethroids in eastern Jamaica can possibly be attributed to the unregulated overuse of household or animal-care products considering that these mosquitoes are usually found in close proximity to human dwellings or possibly from agricultural spraying.

Reduced susceptibility to pyrethroid in *Aedes* mosquitoes is widespread and has been reported in multiple countries [34], inclusive of Jamaica [14]. As part of the WHO global plan for insecticide resistance management in mosquitoes, stakeholders are being urged to improve their knowledge of insecticide resistance in mosquitoes by conducting routine surveillance, utilizing multiple entomological tools to determine prevalence and changes in the levels of insecticide resistance and to ensure that the tools available for vector management remain effective for current and future use [29]. Owing to the low impact of the pyrethroids tested at the baseline concentrations on the *Ae*. *aegypti* populations from eastern Jamaica, higher concentrations designated by WHO as intensity assays were also assessed. Of the four pyrethroids tested at varying (5x and 10x) concentrations, deltamethrin at 10x concentration (0.3%) was toxic to the St. Catherine population that resulted in > 90% (94.90 ± 0.34%) knockdown within 1 hour of exposure and 98.95 ± 0.01% mortality (*p* <0.01) at 24 hours post exposure (Fig 3). The increase in deltamethrin from 0.03 to 0.3% resulted in a seven-fold increase in susceptibility, from 13.46 ± 3.55 at 1-hour exposure (Fig 3). The population exposed to the 10x concentration was susceptible with 98% mortality, well within the parameter recommended by the WHO guidelines [9] for vector management of *Ae*. *aegypti*.

The impact of organophosphates tested on the mosquitoes from eastern Jamaica was minimal (Fig 4). The study showed that mosquitoes from eastern Jamaica were very tolerant to the organophosphates Malathion and pirimiphos. The results are not surprising considering the long documented use of organophosphates for vector control [3] and agricultural practices [41] on the island, for more than forty-five years. The development of resistance to Malathion in *Ae*. *aegypti* collected from St. Andrew, Jamaica had been reported as early as 1995 [2], and its use, though sporadic, was continued [3].

Bendiocarb, on the other hand, appeared to be an effective toxin against mosquitoes from most eastern parishes of Jamaica. The test for susceptibility to bendiocarb was conducted using the CDC bottle bioassays at the diagnostic concentration and exposure time (Fig 5). It was noticed that some of the tested mosquitoes from KSA, St. Catherine and St. Mary were larger, weighing on average ≥ 1.1 mg and took longer than the CDC recommended time of 30 minutes [12] to die. Studies have suggested that weight difference in *Aedes* spp. can account for toxicity differences [42, 43]. Although there were no significant differences in mortality at 12.5μg bendiocarb for the exposure time of 30 minutes amongst the population, there was an observable difference in time to death and body size in some of the populations. The dosage of bendiocarb may need adjusting to accommodate for size difference to be considered an overall effective toxin for the populations from Eastern Jamaica. Mechanistic resistance to carbamates utilizes the same pathway to that used for organophosphate resistance [12, 15, 16]. Although there is no history of use of bendiocarb in Jamaica’s vector control [2, 3], it may also be speculated that the slow time to death observed in some populations, especially KSA, may also be owed to the prevention of bendiocarb binding to its target site in the mosquitoes, because of a pre-established mechanism of resistance to organophosphates. In a previous study on *Ae*. *aegypti* collected from St. Andrew, Jamaica, elevated alpha, beta and *p*-nitro phenyl acetate esterase were observed in the populations [14]. Further analysis needs to be carried out to confirm the reason for the slow responsive time to death observed for bendiocarb.

The presence of mutations in *vgsc* were assessed to characterize possible mechanism for pyrethroid resistance. The frequency of resistant Ile1016 allele was high (62%; see Table 2) in the St. Catherine population. High frequency of *V1016I* mutation in the *vgsc* has been shown to be associated with reduced pyrethroid sensitivity [21], an observance that is not novel to Jamaica [14] or the Americas [24, 33, 44, 45]. From the study, it is possible that this mutation could also be present in the other populations established, owing to the observed similarities in behavioural response to pyrethroid throughout all populations tested. Mutations for *kdr1011*, also associated with pyrethroid resistance [46], and detected at low frequencies in some areas of Latin America [33, 46] was not observed in the St. Catherine population.

The present study shows that *Ae*. *aegypti* from the eastern parishes of Jamaica exhibit tolerance to type I, type II and non-ester pyrethroids and the organophosphates malathion and pirimiphos-methyl, but appear susceptible to the carbamate bendiocarb. Evaluation of the level of resistance to pyrethroids in the St. Catherine population showed strong resistance, whereby only the exposure to deltamethrin at concentration 10x, effectively resulted in death > 98% at 24 hours. The mechanism of pyrethroid resistance in this population may be contributed to the presence of the *kdr* mutation *V1016I*. Based on other studies in St. Andrew, Jamaica [14], the reduced response to pyrethroid may also be associated with elevation of oxidases known to metabolise pyrethroids. The regained susceptibility using 0.3% deltamethrin has operational significance considering that insecticides used for field application are more concentrated. However, unregulated and frequent use of pyrethroids have been shown to select for resistant genes that reduces the efficacy of field application [46]. Management of these insecticides as well as field efficacy evaluations of the insecticides are therefore necessary to reduce resistant-selective pressures and to achieve successful vector control campaigns in the field.

## Conclusion

Nationwide strategies to reduce *Aedes* population and, by extension, mitigate the spread of their diseases are limited by the availability of tools used in vector abatement programs. Most programs are heavily dependent on the use of chemical insecticides to reduce populations.

From the study, it is safe to say that mosquitoes from the eastern parishes of Jamaica are strongly resistant to pyrethroids and, as such, pyrethroids used in the *Aedes* management strategies should be limited or even restricted, including the possibility of regulations being enforced to minimize the use of pyrethroid-containing products, considering that pyrethroid use for mosquito management strategies in Jamaica is relatively new. It is advised that while enforcing strict pyrethroid regulations, continued pyrethroid susceptibility tests should be conducted in the *Aedes* mosquito populations in eastern Jamaica to assess possible reversal in resistance. The results of the current study, particularly the intensity assays, should be used as a baseline to denote either increase in- or reversal of- resistance to inform field operational efficacy of insecticides. It is also recommended that other tools, such as community engagement in mosquito reduction strategies, larvicide campaigns, and the use of other chemicals, such as bendiocarb, be incorporated as innovative tools in the routine vector management strategies in the eastern sections of Jamaica. Furthermore, manufacture guidelines should be adhered to when using chemical toxins in *Aedes* control strategies in an effort to prevent observance of resistance to other chemical toxins.

Our findings clearly highlight the need for continuous and widespread insecticide-susceptibility monitoring in *Aedes* vectors using standard and internationally accepted procedures.

## Supporting information

S1 Table Suceptibility studies for adult *Aedes aegypti* collected from eastern parishes of Jamaica. Mortality data for each insecticide tested.
(XLSX)

S2 Table Table showing *kdr* gene frequency information for each pyrethroid tested in adult *Aedes aegypti* collected from St. Catherine, Jamaica. (XLSX)
